# Prognostic Value of Serum Galectin-3 in Chronic Heart Failure: A Meta-Analysis

**DOI:** 10.3389/fcvm.2022.783707

**Published:** 2022-02-18

**Authors:** Zhendong Cheng, Kefeng Cai, Chaoxian Xu, Qiong Zhan, Xingbo Xu, Dingli Xu, Qingchun Zeng

**Affiliations:** ^1^State Key Laboratory of Organ Failure Research, Department of Cardiology, Nanfang Hospital, Southern Medical University, Guangzhou, China; ^2^Department of Cardiology, The Second Affiliated Hospital of Fujian Medical University, Quanzhou, China; ^3^Guangdong Provincial Key Laboratory of Shock and Microcirculation, Southern Medical University, Guangzhou, China; ^4^Bioland Laboratory (Guangzhou Regenerative Medicine and Health Guangdong Laboratory), Guangzhou, China; ^5^Department of Cardiology and Pneumology, University Medical Center of Göttingen, Georg-August-University, Göttingen, Germany

**Keywords:** galectin-3, chronic heart failure, all cause death, cardiovascular death, meta-analysis

## Abstract

**Objective:**

To evaluate the association between serum galectin-3 and all-cause death (ACD) and cardiovascular death (CVD) in patients with chronic heart failure (CHF).

**Methods:**

The PubMed and Embase databases and Clinical Trials Registry (www.clinicaltrials.gov) were searched for studies with data on serum galectin-3 and ACD and CVD in CHF patients. The hazard ratios (HRs) of ACD and CVD were calculated and presented with 95% CIs. HRs were pooled using fixed effects or random effects models when appropriate. Sensitivity analysis, meta-regression and subgroup analysis were applied to find the origin of heterogeneity. Visual inspection of Begg's funnel plot and Egger's test were performed to assess the possibility publication bias.

**Results:**

Pooled data included the results from 6,440 patients from 12 studies in the meta-analysis. Higher serum galectin-3 was associated with a higher risk of ACD (HR, 1.38; 95% CI, 1.14–1.67) and CVD (HR, 1.13; 95% CI, 1.02–1.25) in CHF patients. In the subgroup analyses, higher serum galectin-3 was associated with an increased risk of ACD in all subgroups. The pooled HR of the shorter follow-up group (1.78; 95% CI, 1.50–2.11) was significantly higher than the pooled HR of the longer follow-up group (1.15; 95% CI, 1.05–1.25). Sensitivity analysis of eliminating one study in each turn indicated that Koukoui et al.'s study had the largest influence on the risk of all-cause death. All-cause death publication bias was not detected (Pr>|z| = 0.35 for Begg's test and P>|t| = 0.15 for Egger's test).

**Conclusions:**

Serum galectin-3 has prognostic value of both all-cause death and cardiovascular death in CHF. Serum galectin-3 could be useful for risk classification in patients with CHF.

**Systematic Review Registration:**

https://www.crd.york.ac.uk/prospero/display_record.php?RecordID=193399.

## Introduction

Chronic heart failure (CHF) is a common clinical syndrome in cardiology with high morbidity and mortality worldwide (1). Despite significant advances in the diagnosis and treatment, the prognosis for patients with CHF remains poor. About 17.9 million people die from cardiovascular disease each year, of which 9.6 percent are due to heart failure (2). Lack of precise, repeatable and effective prognostic biomarkers may be one of the reasons for poor prognosis in patients with heart failure. Thus, we urgently need to find novel different prognostic biomarkers, which may be able to increase new pathophysiological insight and to guide the precise preventive and therapeutic strategies in CHF patients.

Myocardial fibrosis is a major determinant of clinical outcomes in patients with CHF. Fibrosis markers, such as galectin-3, soluble suppression of tumorigenicity 2 (sST2), human epididymis protein 4 (HE4), metalloproteinases (TIMP)-1, and matrix metallopeptidase (MMP)-9 have been evaluated in HF (3–13). Galectin-3, a β-galactoside–binding lectin mainly secreted by activated macrophages, is associated with myocardial fibrosis and the progression of HF (5, 14, 15). Galectin-3, as a marker of myocardial fibrosis, has been included in the European and American HF guidelines, with a class IIb recommendation (16, 17). However, galectin-3 is not widely used in clinical practice. In an earlier meta-analysis, elevated levels of galectin-3 were found to be associated with mortality in CHF patients (18). However, some recently published studies that were not included in that meta-analysis have reported that the association of galectin-3 with all-cause death (ACD) and cardiovascular death (CVD) are inconsistent (19–21). Therefore, in this study, a meta-analysis was performed to systematically evaluate the prognostic role of serum galectin-3 in patients with CHF.

## Materials and Methods

Our meta-analysis was performed followed the Preferred Reporting Items of PRISMA statement. We registered this meta-analysis in the PROSPERO database (CRD42020193399).

### Search Strategy

We conducted the meta-analysis according to the Meta-analysis of Observational Studies in Epidemiology Group (22). We performed a comprehensive literature search of studies in the PubMed and Embase databases Clinical Trials Registry (www.Clinicaltrials.gov) up to April 10, 2020. Two search themes were combined using the Boolean operator “and.” The first theme was heart failure, combined exploded versions of medical subject headings (MeSH) *heart failure, cardiac failure, heart decompensation, myocardial failure, congestive heart failure*. The second theme, *galectin 3*, combined exploded versions of MeSH terms *galectin 3, galectin-3*, or *Gal-3*. The exact search string was used for pubmed and modified to suit Embase database: (((“Heart Failure”[Mesh]) OR ((((Cardiac Failure) OR Heart Decompensation) OR Myocardial Failure) OR Congestive Heart Failure))) AND ((“Galectin 3”[Mesh]) OR ((galectin-3) OR Gal-3)).

### Literature Inclusion and Exclusion Criteria

Studies that met the following criteria were included in the analysis: (1) enrollment of outpatients with CHF (either HFrEF or HFpEF); (2) follow-up studies with adult participants (aged ≥ 18 years); (3) serum galectin-3 was measured; (4) the relationship between galectin-3 and all-cause death (ACD) was reported, possibly, also for cardiovascular death (CVD); (5) multivariable adjusted hazard ratio (HR) and the corresponding 95% confidence interval (CI) were available; (6) English language. Exclusion criteria: (1) studies on patients with end-stage HF; (2) studies that cannot provide valid data for estimating HR and 95% CI; (3) only unadjusted risks were provided for associated outcomes; (4) duplicate data or analyses.

### Data Extraction and Quality Assessment

On the basis of the predefined criteria, two independent authors (Zhendong Cheng and Kefeng Cai) evaluated and screened the candidate studies. When a disagreement arised, two authors reached a consensus by discussing it with the third author (Chaoxiang Xu). The following basic information were recorded each study: first author, country, year of publication, sample size, percentage of males, mean age, follow-up time, LVEF of the participants, and plasma galectin-3 levels. The quality of each included study was assessed using the Newcastle-Ottawa Scale (NOS) by two independent authors (Zhendong Cheng and Kefeng Cai) (23). The NOS assesses studies according to 9 issues. The 9 iconic questions were assessed as “Yes” (clear fit), “Unclear,” and “No” (not meeting the requests). According to the evalution issues, biases were classified as high risk, unclear, and low risk.

### Statistical Analysis

The primary end point measure was the risk of all-cause death (ACD) associated with serum galectin-3. The secondary end point measure was the risk of cardiovascular death (CVD) associated with serum galectin-3. From each study, multivariate adjusted outcome data (hazard ratios (HRs) and 95%CIs) for all-cause death (ACD) and cardiovascular death (CVD) were recorded for analysis (24). The heterogeneity of the included studies was estimated by Cochran's Q test and Higgins I-squared statistics. A *P* < 0.10 or *I*^2^ >50% indicated existence of significant heterogeneity.

The pooled HRs and 95% CIs were calculated by using both the random-effects and fixed-effects model. If there was no or low heterogeneity, the fixed effects model was used, Otherwise a random effects model was adopted. To explore the origin of heterogeneity, we conducted subgroup analysis of the primary end point according to mean age (<65 vs. ≥65 years); sample size (<300 vs. ≥300); follow-up period (<40 vs. ≥40 months); publication year (<2015 vs. ≥2015). Meta-regression analysis was applied to explore the potential impact of population characteristics on primary outcome. In addition, a sensitivity analysis was conducted to explore the heterogeneity of different studies. The pooled HR was recalculated by omitting 1 study at a time. We evaluated publication bias for all cause death (ACD) by using Begg's funnel plot and Egger's test (25).

All the statistical analyses were conducted using STATA version 12.0 (StataCorp LP, College Station, TX, USA). The graphic displays of Newcastle-Ottawa Scale (NOS) assessment were performed by RevMan 5.2 (The Cochrane Collaboration, Copenhagen, Denmark). All *p* values were two tailed with a statistical significance level of 0.05.

## Results

### Literature Search Results and Characteristics

**Figure 1:**
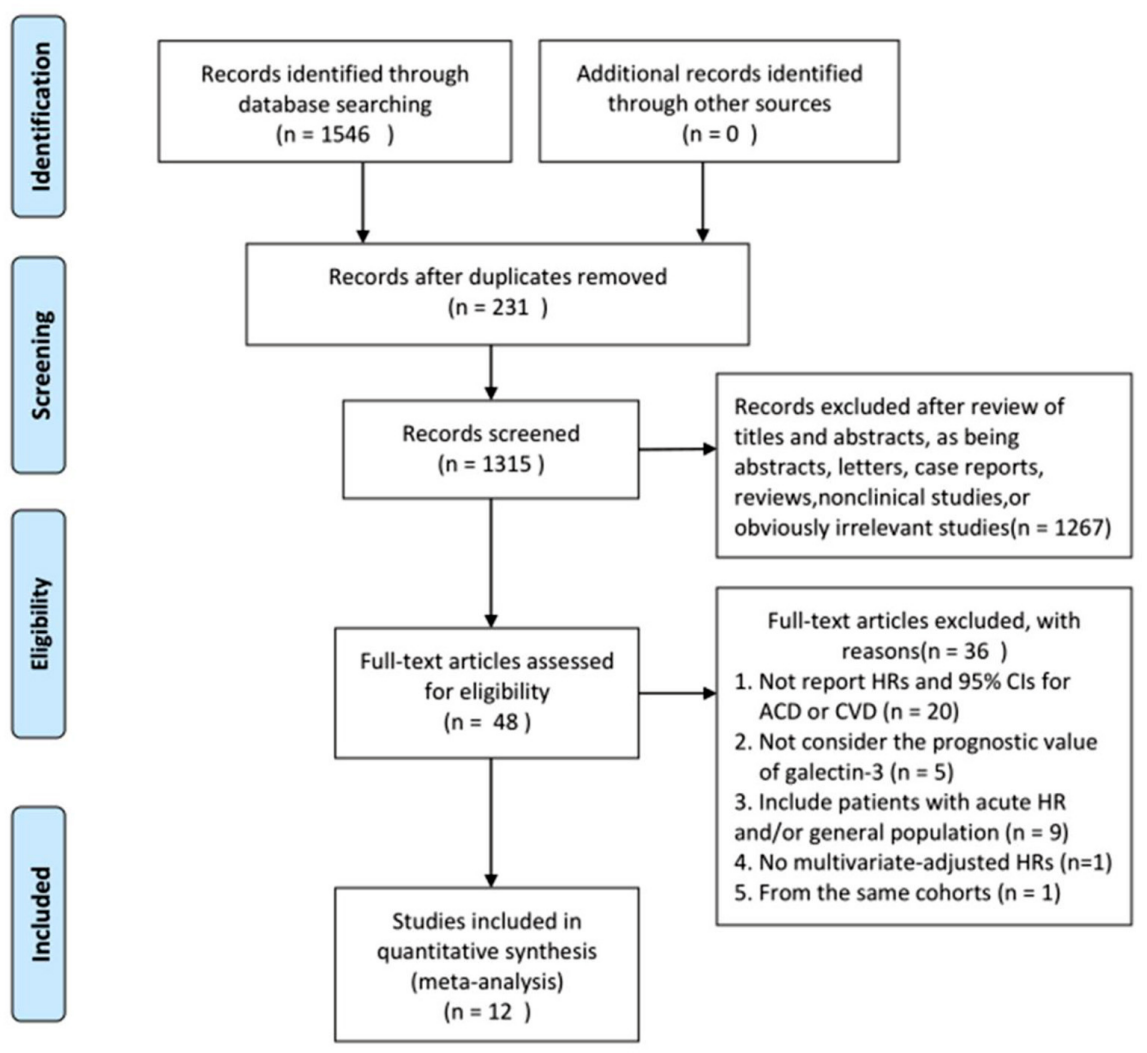
Flowchart of study selection. HRs, hazard ratios; CIs, confidence intervals; ACD, all cause death; CVD, cardiovascular death.

**Figure 2:**
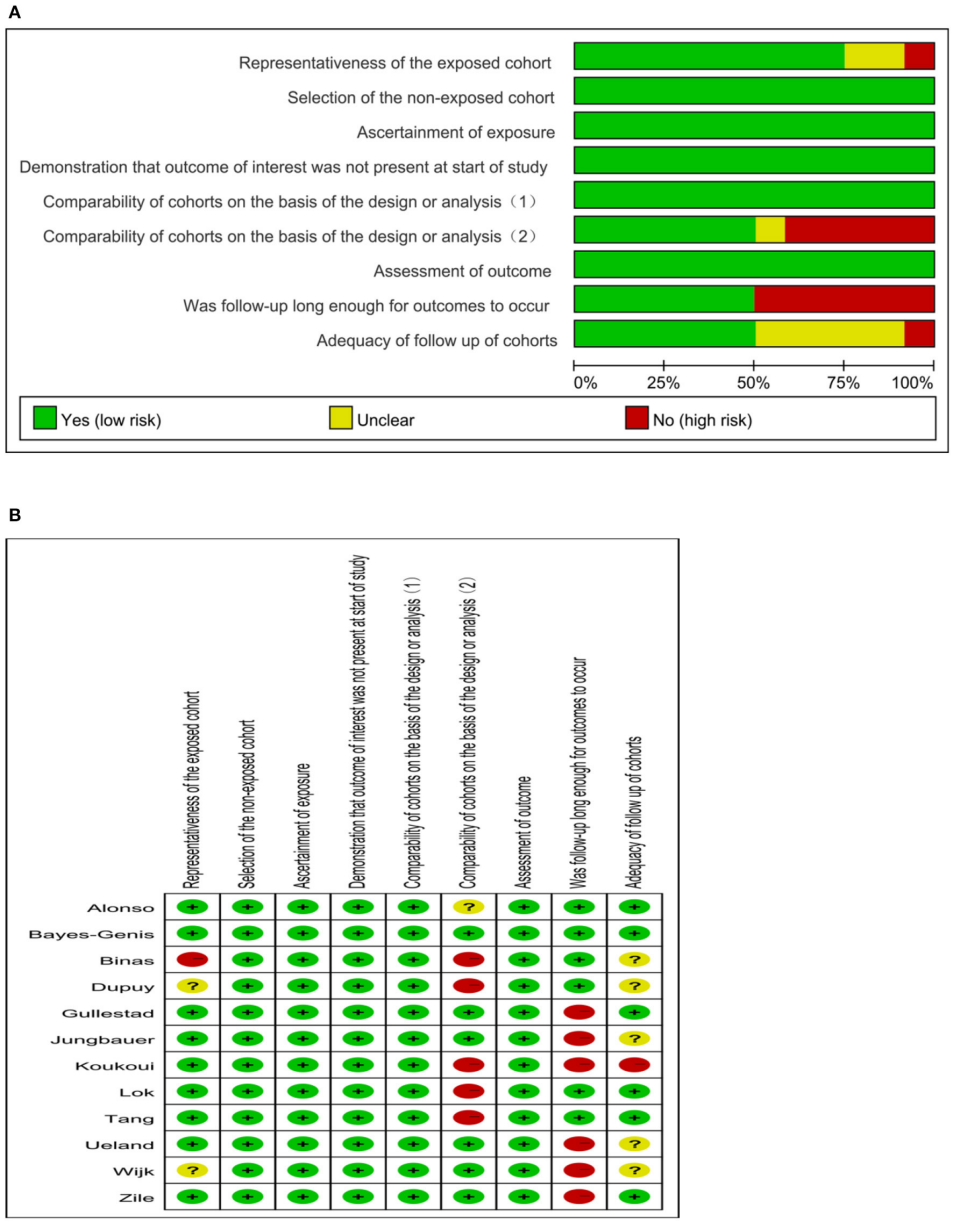
Quality evaluation of the eligible studies. **(A)** Review authors' judgments presented as percentages across included studies; **(B)** Review authors' judgements about each domain for each included study.

The search process is summarized in Figure 1. Initially, in the primary search from the PubMed and Embase databases, we retrieved a total of 1,546 articles. After meticulous inspection of the articles. Twelve studies involving 6,440 participants were selected for our meta-analysis (10, 19–21, 26–33). There were no disagreements on the inclusion of studies among reviewers. The basic patient characteristics of the included studies are shown in Table 1. Of the 12 studies, 10 studies enrolled only patients with reduced LVEF (19–21, 26–28, 30–33), whereas 2 studies also considered patients with preserved LVEF (10, 29). HRs and 95% CIs were provided directly in all studies, and HRs were calculated via multivariate analysis (10, 19–21, 26–33). Five of these studies enrolled >300 patients (21, 28, 30, 31, 33) and 7 studies had <300 patients (10, 19, 20, 26, 27, 29, 32). All studies were from Western countries, 10 were from Europe (10, 19–21, 27–32), one was from the United States (26), and one was from Europe, the United States and Canada (33). The mean age of the patients varied from 50 to 76 years, and the duration of follow up varied from 8 to 116 months. All studies included both sexes, with the proportion of men amounting to 76.9%. Four reported all-cause and cardiovascular death (19, 21, 28, 30), 1 reported only cardiovascular death (33), and 7 reported only all-cause death (10, 20, 26, 27, 29, 31, 32). Therefore, there were 11 and 5 studies for analyses of all-cause and cardiovascular death, respectively. Figure 2 summarizes the Newcastle-Ottawa Scale (NOS) assessments of the eligible studies.

**Table 1:** Characteristics of the studies included in meta-analysis.

**References**	**Country**	**Sample size (*n*)**	**Age (years)**	**Male (%)**	**HFrEF (%)**	**LVEF (%)**	**Follow-up time (month)**	**Galectin-3 assay**	**Galectin-3 levels (ng/ml)**	**Outcomes reported**
Tang et al. (26)	United States	133	57 ± 13	74	100	26 ± 6	60	ELISA Bender MedSystems	13.9 (12.1–16.9)	ACD
Ueland et al. (27)	Norway	168	56 ± 12	78	100	31 ± 14	35 ± 16	ELISA BG Medicine	15.3 (median)	ACD
Gullestad et al. (28)	Norway; Sweden; United Kingdom	1,462	72 ± 7	76	100	32 ± 7	32	ELISA BG Medicine	T1: <16.7; T2:16.7–21.6; T3: <21.6	ACD; CVD
Lok et al. (10)	Netherlands	232	71 ± 0.6	73	97	NA	104 ± 12	ELISA BG Medicine	17.6 (13.3–21.4)	ACD
Jungbauer et al. (29)	Germany	149	62 ± 11	81	97	NA	23 (17–30)	ELISA BG Medicine	35.1 (33.1–37.6)	ACD
Bayes-Genis et al. (30)	Spain	876	70 (61–77)	72	100	34 (26–43)	60	ELFA BioMerieux	16.5 (12.6–22.7)	ACD; CVD
Koukoui et al. (20)	Netherlands	202	58 ± 13	77	100	30 (26–34)	14 (6–20)	bioMérieux, Marcy l'Etoile, France	14 (9.9–19.8)	ACD
Sanders-van Wijk et al. (31)	Italy	631	66 ± 11	82	100	31 ± 7	18	ELISA BG Medicine	18.8 (15.5–24.1)	ACD
Alonso et al. (21)	Netherlands	385	68 ± 10	69	100	33 ± 13	59 ± 34	ELFA BioMerieux	17.4 (14–23.4)	ACD; CVD
Binas et al. (32)	Germany	262	50 ± 13	75	100	30 ± 8	47 (12–91)	ELISA kit (R&D Systems)	4.8 ± 2.3	ACD
Dupuy et al. (19)	France	164	76 (66–82)	69	100	35 (25–45)	42 (12–47)	ELFA BioMerieux	19.8 (14.23–29.73)	ACD; CVD
Zile et al. (33)	United States; Canada; Sweden; United Kingdom	1,776	67 ± 10	81	100	NA	8	ELISA BG Medicine	17.1 (13.9–21.2)	CVD

*HFrEF, Heart failure with reduced ejection fraction; LVEF, left ventricular ejection fraction; NA, not available; NOS score, the Newcastle-Ottawa Scale score; ACD, all cause death; CVD, cardiovascular death*.

### Galectin-3 and ACD

**Figure 3:**
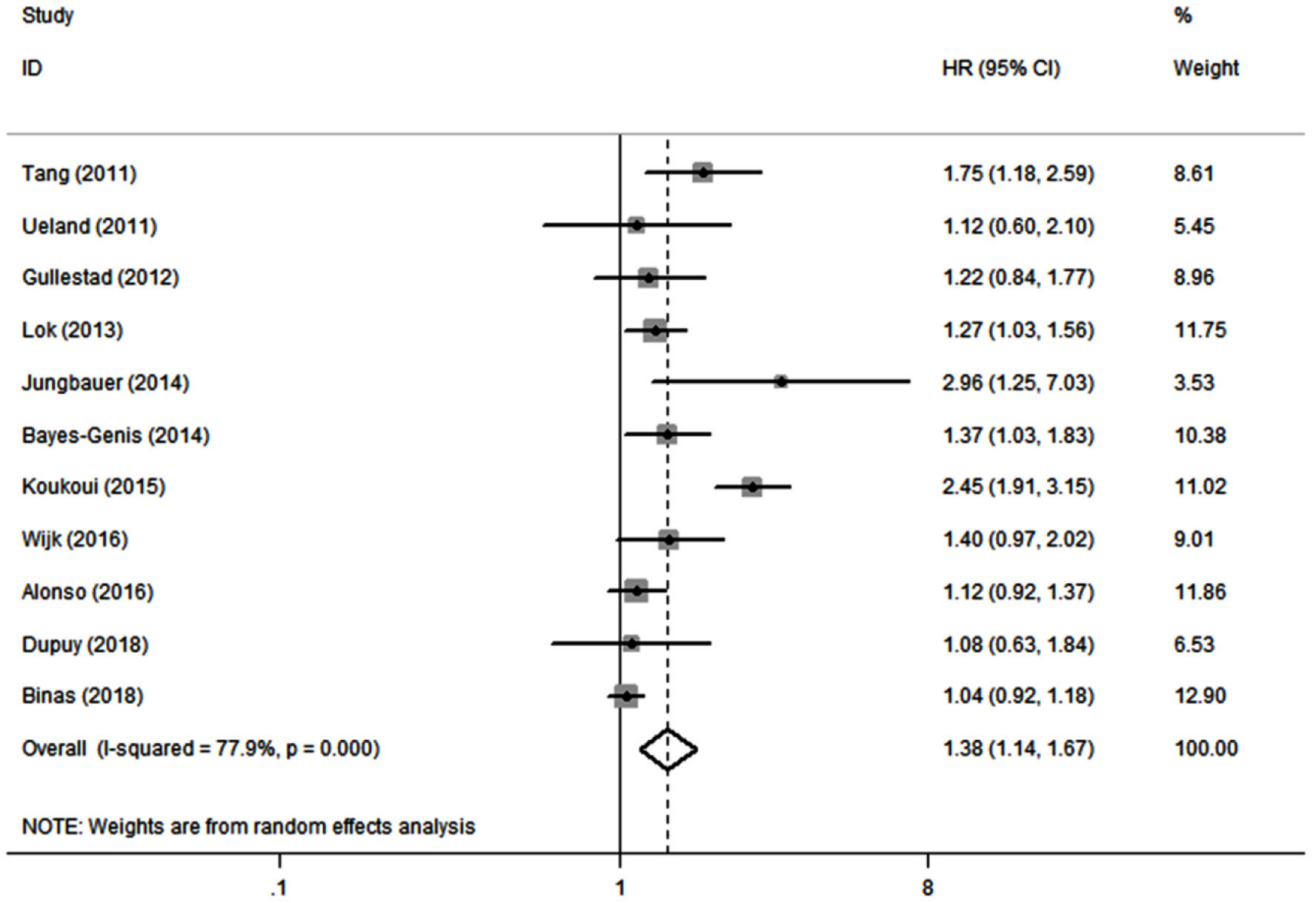
Meta-analysis of the association between galectin-3 and risk of ACD in CHF patients. Results are presented as individual and pooled HR, and 95% CI.

Eleven studies (10, 19–21, 26–32) evaluated the association between serum galectin-3 and the risk of ACD in CHF patients. Because there was significant heterogeneity (*I*^2^ = 77.9%, *P* < 0.1), a random effects model was adopted. Our results showed that elevated serum galectin-3 was associated with a higher risk of ACD in CHF (HR, 1.38; 95% CI, 1.14–1.67; Figure 3).

### Galectin-3 and CVD

**Figure 4:**
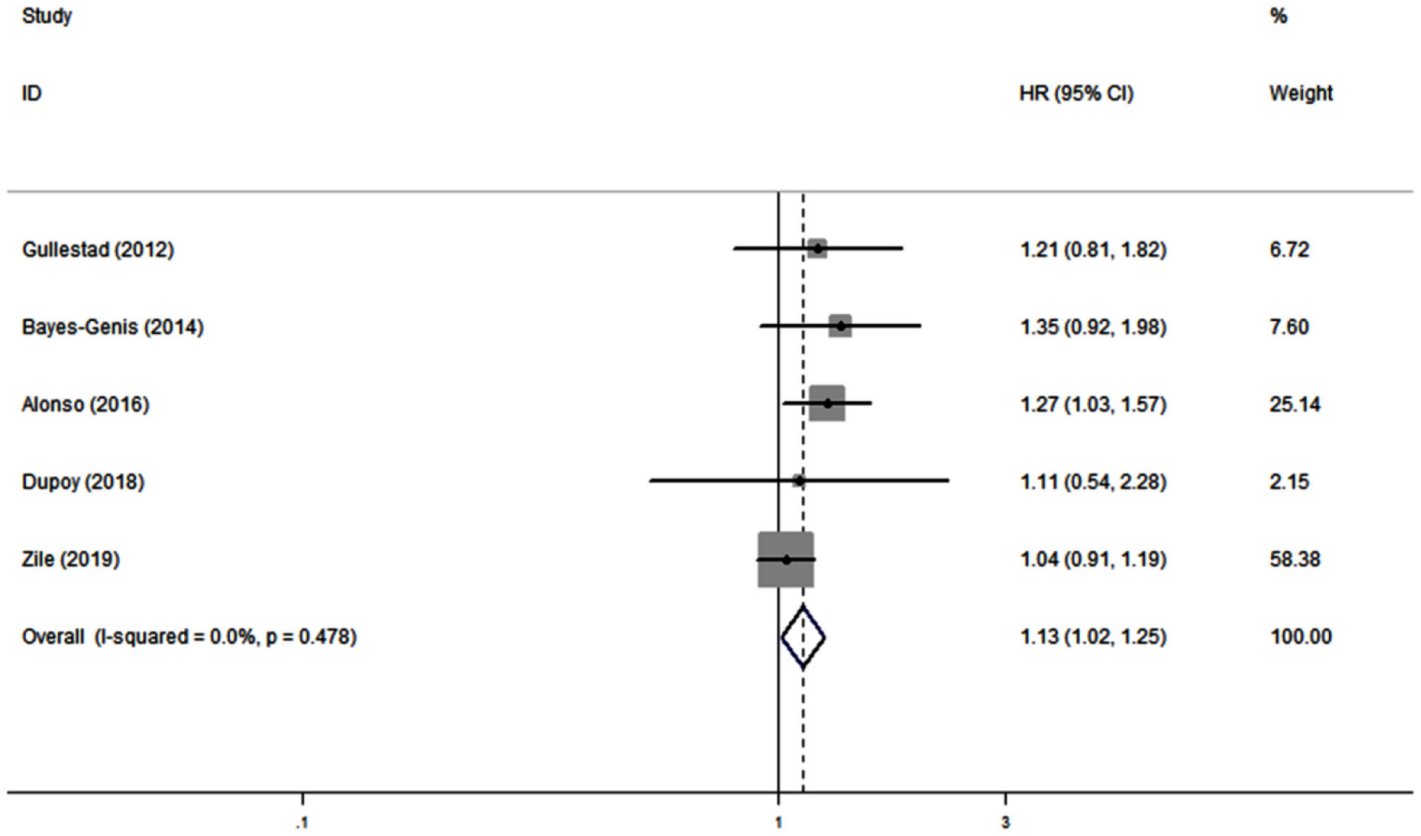
Meta-analysis of the association between galectin-3 and risk of CVD in CHF patients. Results are presented as individual and pooled HR, and 95% CI.

Five studies (19, 21, 28, 30, 33) reported the association between serum galectin-3 and the risk of CVD in CHF. There was no substantial heterogeneity (*I*^2^ = 0.0%, *P* = 0.478); therefore, a fixed effects model was adopted. Our results showed that elevated serum galectin-3 was associated with a higher risk of CVD in CHF (HR, 1.13; 95% CI, 1.02–1.25; Figure 4).

### Subgroup Analyses, Meta-Regression Analyses, and Sensitivity Analyses

In the subgroup analyses, elevated serum galectin-3 was associated with an increased risk of ACD in all subgroups, with analyses conducted according to participant age, follow-up duration, participant number, and publication year of the original study (Table 2). The increased risk was more evident in the shorter follow-up (≤ 40 months) subgroup. The pooled HR of shorter follow-up (1.78; 95% CI, 1.50–2.11) was higher than the pooled HR of longer follow-up (1.15; 95% CI, 1.05–1.25). Due to the limited available studies, we did not perform a subgroup analysis of CVD.

**Table 2:** Subgroup analyses of the association between galectin-3 and risk of ACD in CHF patients.

**Subgroup**	**Number of studies**	**HRs (95% CIs)**	***P* value for heterogeneity**	***I*^2^ value**	***P* value between groups**
**Mean age**					
<65	5	1.28 (1.15,1.42)	0.00	90.7	0.648
≥65	6	1.23 (1.10,1.38)	0.822	0.0	
**Sample size**					
<300	7	1.27 (1.16,1.39)	0.000	86.1	0.685
≥300	4	1.23 (1.07,1.41)	0.6	0.0	
**Follow-up duration**					
<40	5	1.78 (1.50,2.11)	0.004	73.9	0.00
≥40	6	1.15 (1.05,1.25)	0.095	46.6	
**Publication year of the original study**					
<2015	6	1.36 (1.18,1.56)	0.323	14.2	0.182
≥2015	5	1.21 (1.11,1.33)	0.00	89.4	

In 11 studies that reported the risk of ACD, meta-regression analysis showed no significant associations among study characteristics (participant age, follow-up duration, participant number, and publication year of the original study) and risk of ACD (all *P* > 0.05).

**Figure 5:**
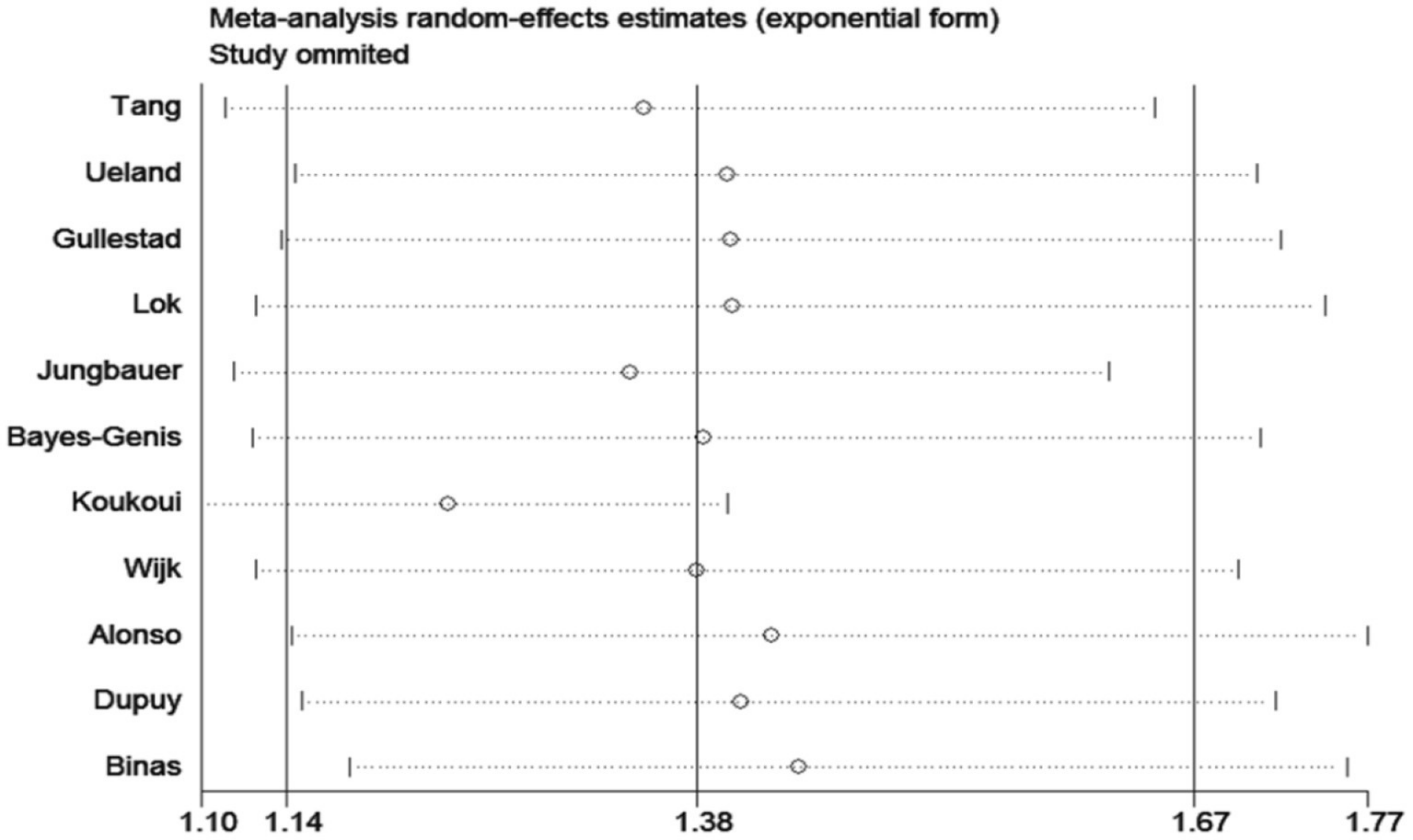
Sensitivity analysis [Removing each study (author's name) one at a time].

The sensitivity analyses confirmed that the association between ACD and galectin-3 did not change with the use of random effects models or fixed effects models for the meta-analysis. A sensitivity analysis of omitting one study at a time revealed that Koukoui et al. study (20) had the largest impact on the overall results: the pooled HR omitting this study was 1.24 (95% CI, 1.09–1.40) (Figure 5).

### Analysis of Publication Bias

**Figure 6:**
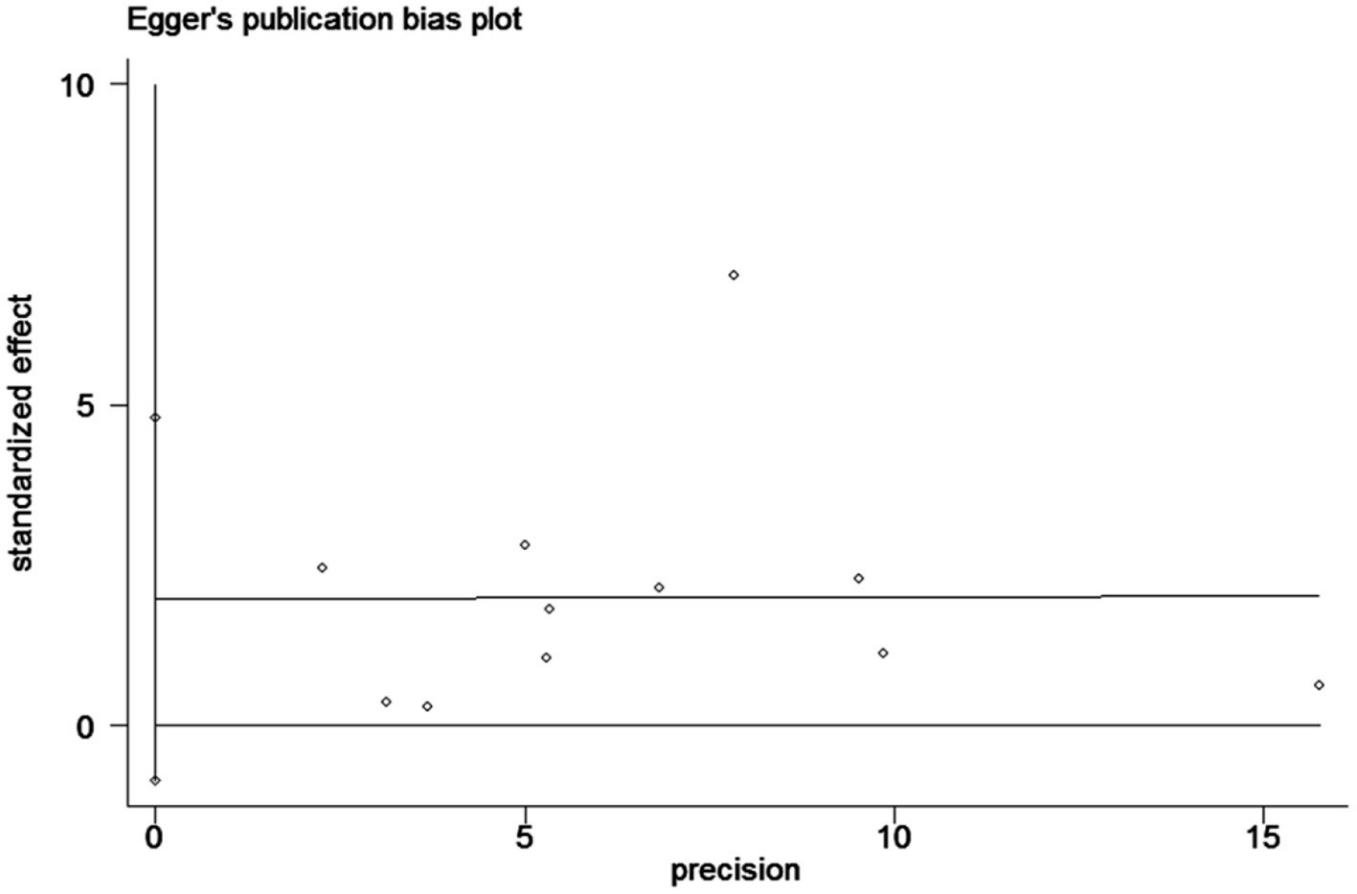
The funnel plot of publication bias.

Begg's tests and Egger's tests (Figure 6) were conducted to assess publication bias. ACD publication bias was not detected (P>|t| = 0.15 for Egger's test and Pr>|z| = 0.35 for Begg's test).

## Discussion

In this meta-analysis, we combined the outcomes of 6,440 CHF patients from 12 individual studies. The aggregated results indicated that serum galectin-3 is an independent predictor of ACD and CVD in CHF patients. Eleven studies reported an association between serum galectin-3 and the risk of ACD in CHF patients, with a pooled HR of 1.38 (95% CI: 1.14–1.67, Figure 3). Five studies presented data on the association between serum galectin-3 and the risk of CVD, with a pooled HR of 1.13 (95% CI, 1.02–1.25; Figure 4). Taking our aggregate results into consideration, serum galectin-3 may be a strong and independent biomarker in the prognosis of CHF.

The result of ACD is consistent with a previous meta-analysis published in 2015 (34), which is the current analysis concerning the prognostic value of serum galectin-3 in patients with CHF. However, only articles published before 2014 were enrolled, and this results necessary to be updated and validated. Moreover, although there was significant heterogeneity (*I*^2^ = 80), no further analyses were performed. Compared with this meta-analyses, our study provides significant strengths. We evaluated the predictive role of serum galectin-3 for ACD and CVD in CHF patients. Moreover, sensitivity analyses, meta-regression analyses and subgroup analyses were applied to search for the origin of heterogeneity.

BNP and NT-proBNP are the most well-established biomarkers used in evaluating prognosis of patients with heart failure, which are included in the current guideline and widely used in clinical practice (16, 17). Novel biomarkers in HF may supplement the traditional ones (BNP and NT-proBNP) routinely used by providing additional prognostic, or stratification utility, and so optimizing the treatment of patients. Galectin-3 and sST2 are the only novel HF biomarkers that have been included in the European and American HF guidelines, with a class II b recommendation (16, 17). However, their clinical value is still uncertain.

Galectin-3, a chimeara-type β-galactoside binding lectin, is a crucial molecule in cardiac fibrosis (5, 35). Galectin-3 not only activates multiple profibrotic factors, facilitates proliferation and transformation of fibroblasts, and mediates the production of collagen (36–39). It can also stimulates macrophages to engulf apoptotic cells and cellular debris (40, 41). Animal studies have shown that galectin-3 is involved in cardiac re-modeling, and inhibition of the synthesis of galectin-3 by genetic technology or drugs can reduce cardiac remodeling and alleviate myocardial fibrosis (12, 13, 15, 42–44). In human studies, several studies demonstrated a significant prognostic value of galectin-3, independently from BNP/NT-proBNP and other prognostic variables in patients with HF, as well as in the general population (18, 45–47). However, the relationship between galectin-3 levels and HF in previous studies remains under debate. Some recently published studies have found that serum galectin-3 levels were not directly associated with specific cardiac parameters or major adverse cardiac events of CHF (19, 21, 32, 33, 48). A recent study in which endomyocardial biopsies were obtained from HF patients found that myocardial galectin-3 levels were not correlated with plasma galectin-3 levels, and plasma galectin-3 were not associated with cardiac fibrosis (49). The underlying mechanism may be that, except for cardiac strain and cardiomyocyte-specific cell death, galectin-3 is expressed in multiple organs and/or tissues, such as the kidney, gastric cancer, breast cancer, and lung (12, 50–52). From the results of our meta-analysis, higher serum galectin-3 levels were associated with a higher risk of mortality in chronic HF patients. Because HF is a multi-system syndrome affecting many tissues and organs, and galectin-3 is a biomarker not organ-specific but specific for individual pathogenesis, in particular inflammation or fibrosis, it is likely that other organs or tissues could also contribute to increased serum galectin-3 levels. Thus, it is not surprising that they have a strong prognostic value. In the subgroup analyses, elevated serum galectin-3 was associated with an increased risk of ACD in all subgroups. Interestingly the pooled hazard ratio (HR) of the shorter follow-up group (<40 months) was significantly higher than the pooled HR of the longer follow-up group. We think it is associated with higher long-term mortality in patients with heart failure. The 5-year survival rate for heart failure patients is even lower than for some patients with cancer. Therefore, galectin-3 may be more suitable for evaluating the short-term prognosis of patients with chronic heart failure. Our study further confirms that galectin-3 is a significant predictor of ACD and CVD in CHF patients. It would help clinicians to adopt timely prevention and effective therapeutic strategies for CHF patients. Our study provides additional evidence for the development of clinical guidelines.

Some limitations of our meta-analysis should be mentioned. First, there was a high heterogeneity across the included studies in HR for ACD (*I*^2^ = 77.9%, *P* < 0.1), which may result from differences in follow-up time and participant characteristics. While there was moderate to high heterogeneity in many subgroups, the pooled HRs revealed consistent positive correlations in different subgroups. A sensitivity analysis of eliminating one study at a time revealed that Koukoui et al.'s study may be the origin of heterogeneity. Second, most of the included studies in the meta-analysis were of high quality. However, some of the involved studies were *post hoc* analyses in designing, which might affect the quality of the meta-analysis. Third, the involved studies in this meta-analysis were all from Western countries, and most of the participants had HF with reduced left ventricular ejection fraction. Thus, further well-designed studies in a wide range of regions and populations are needed to confirm our conclusions. Finally, this study was limited to English publications only, so publication bias may exist.

In conclusion, this meta-analysis provides evidence that higher serum galectin-3 was independently associated with poor prognosis in CHF patients. Given the high morbidity and mortality rates of CHF, our study has significant clinical and public health importance.

## Data Availability Statement

The raw data supporting the conclusions of this article will be made available by the authors, without undue reservation.

## Author Contributions

ZC, KC, CX, QZ, XX, DX, and QZ was involved in conceiving the design of the meta-analysis, analyzing and interpreting data, or drafting/revising the manuscript. All authors contributed to the article and approved the submitted version.

## Funding

This work was partly supported by the National Natural Science Foundation of China (81770386 and 82070403); the Science and Technology Program of Guangdong Province (2021A0505030031); the Frontier Research Program of Guangzhou Regenerative Medicine and Health Guangdong Laboratory (2018GZR110105001); and the Youth Science and Technology Innovation Talent Program of Guangdong TeZhi plan (2019TQ05Y136).

## Conflict of Interest

The authors declare that the research was conducted in the absence of any commercial or financial relationships that could be construed as a potential conflict of interest.

## Publisher's Note

All claims expressed in this article are solely those of the authors and do not necessarily represent those of their affiliated organizations, or those of the publisher, the editors and the reviewers. Any product that may be evaluated in this article, or claim that may be made by its manufacturer, is not guaranteed or endorsed by the publisher.
