# Supplementary figures and images for: Prognostic Value of Serum Galectin-3 in Chronic Heart Failure: A Meta-Analysis
Source: Front Cardiovasc Med. 2022 Feb 18;9:783707. doi: 10.3389/fcvm.2022.783707 (PMC8894589; doi:10.3389/fcvm.2022.783707)

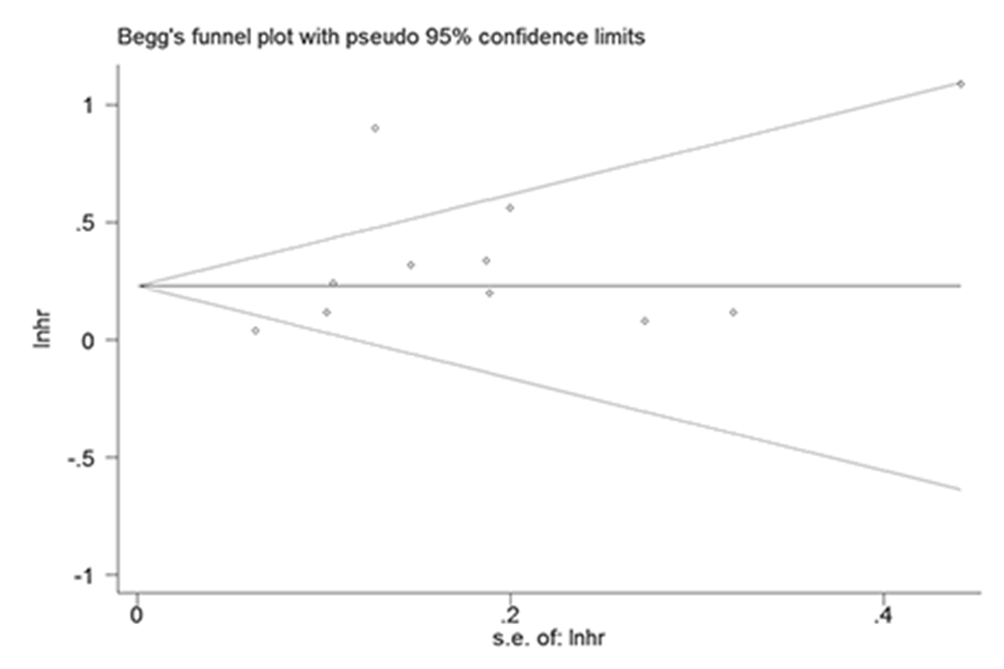

Supplement: Supplementary file 1 [file Data_Sheet_1.ZIP › additional files/Beggs.tif]

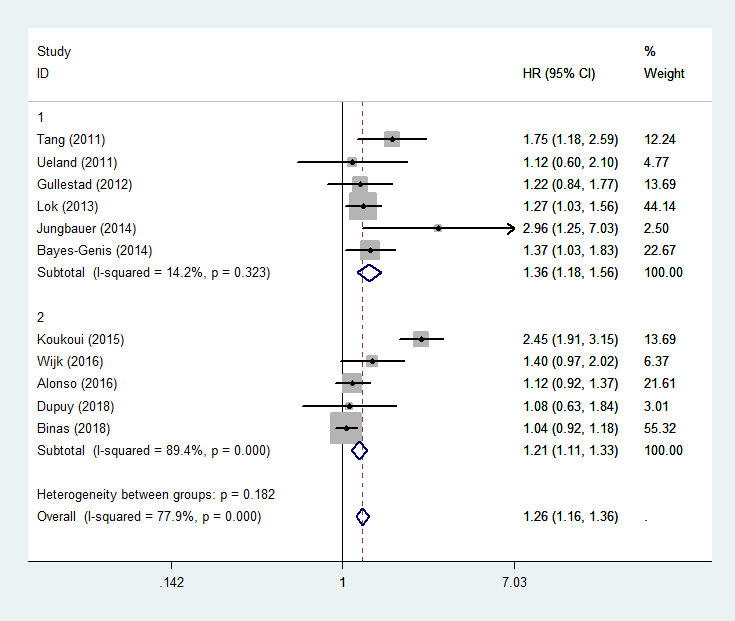

Supplement: Supplementary file 1 [file Data_Sheet_1.ZIP › additional files/Subgroup analyses of the association between galectin-3 and risk of ACD in CHF patients/╖ó▒φ─Ω╤╟╫Θ.tif]

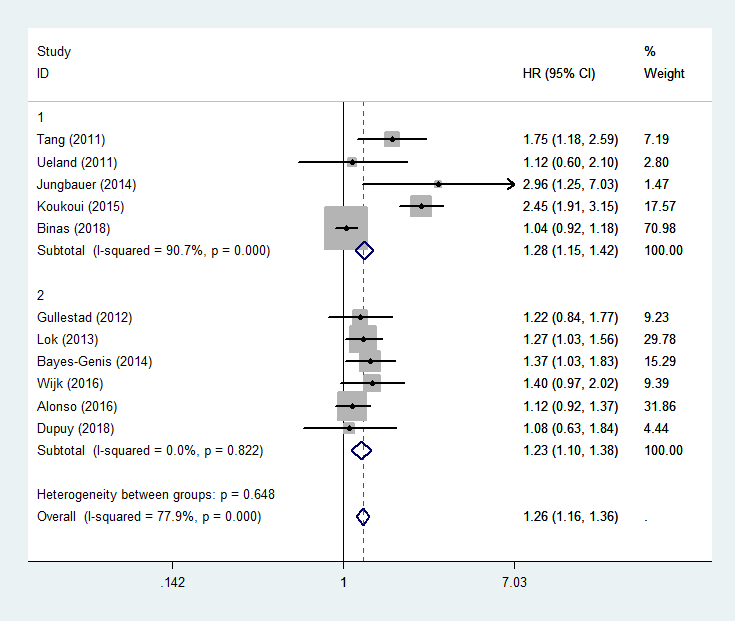

Supplement: Supplementary file 1 [file Data_Sheet_1.ZIP › additional files/Subgroup analyses of the association between galectin-3 and risk of ACD in CHF patients/─Ω┴Σ╤╟╫Θ.tif]

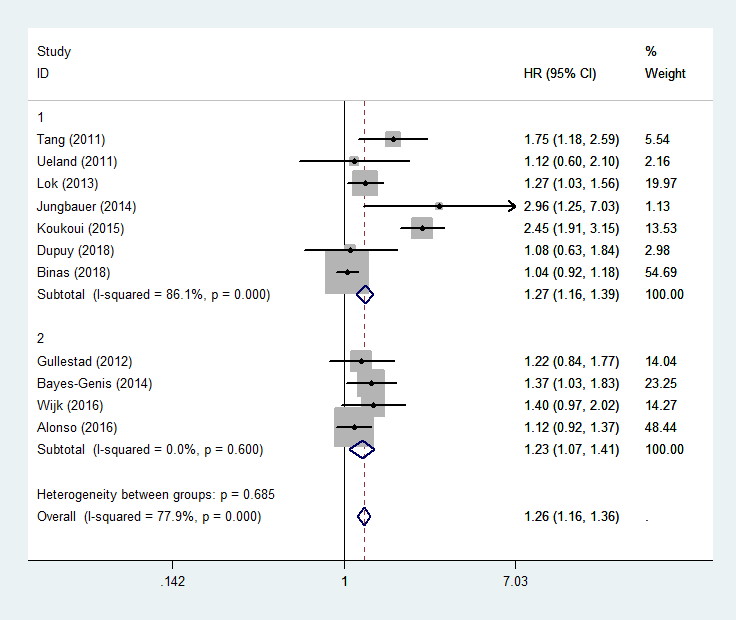

Supplement: Supplementary file 1 [file Data_Sheet_1.ZIP › additional files/Subgroup analyses of the association between galectin-3 and risk of ACD in CHF patients/╤∙▒╛┴┐╤╟╫Θ.tif]

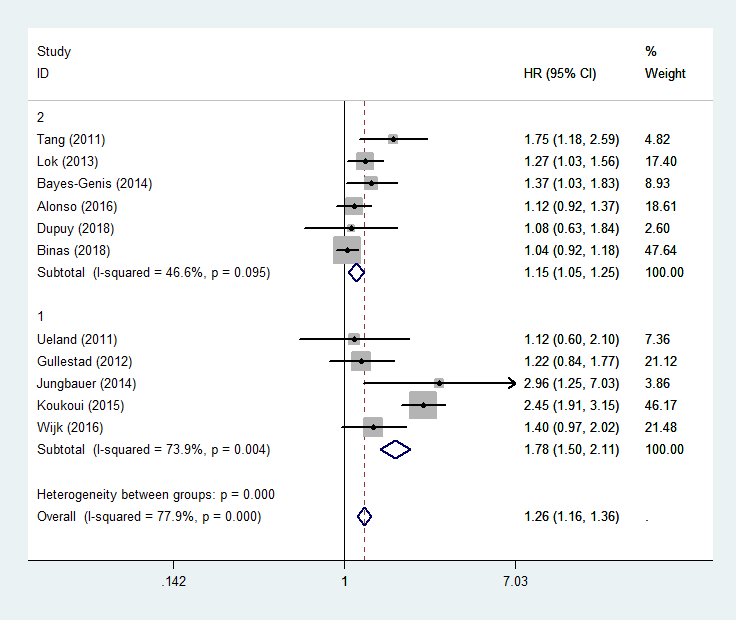

Supplement: Supplementary file 1 [file Data_Sheet_1.ZIP › additional files/Subgroup analyses of the association between galectin-3 and risk of ACD in CHF patients/╦μ╖├╩▒╝Σ╤╟╫Θ.tif]
