# Supplementary material for: Prognostic Value of Serum Galectin-3 in Chronic Heart Failure: A Meta-Analysis
Source: Front Cardiovasc Med. 2022 Feb 18;9:783707. doi: 10.3389/fcvm.2022.783707 (PMC8894589; doi:10.3389/fcvm.2022.783707)
Supplement: Supplementary file 2 [file Data_Sheet_2.PDF]

The exact search string for pubmed

("Heart Failure"[MeSH Terms] OR ("heart"[All Fields] AND "failure"[All Fields]) OR "Heart Failure"[All Fields] OR ("Heart Failure"[MeSH Terms] OR ("heart"[All Fields] AND "failure"[All Fields]) OR "Heart Failure"[All Fields] OR ("cardiac"[All Fields] AND "failure"[All Fields]) OR "cardiac failure"[All Fields]) OR ("Heart Failure"[MeSH Terms] OR ("heart"[All Fields] AND "failure"[All Fields]) OR "Heart Failure"[All Fields] OR ("heart"[All Fields] AND "decompensation"[All Fields]) OR "heart decompensation"[All Fields]) OR ("Heart Failure"[MeSH Terms] OR ("heart"[All Fields] AND "failure"[All Fields]) OR "Heart Failure"[All Fields] OR ("myocardial"[All Fields] AND "failure"[All Fields]) OR "myocardial failure"[All Fields]) OR ("Heart Failure"[MeSH Terms] OR ("heart"[All Fields] AND "failure"[All Fields]) OR "Heart Failure"[All Fields] OR ("congestive"[All Fields] AND "heart"[All Fields] AND "failure"[All Fields]) OR "congestive heart failure"[All Fields]) OR "Heart Failure"[MeSH Terms]) AND ("Galectin 3"[MeSH Terms] OR "Galectin 3"[All Fields] OR ("Galectin 3"[MeSH Terms] OR "Galectin 3"[All Fields]) OR "Gal-3"[All Fields] OR "Galectin 3"[MeSH Terms])

History and Search Details

Search	Actions	Details	Query
#7	•••	>	Search: **((((((Heart Failure) OR (Cardiac Failure)) OR (Heart Decompensation)) OR (Myocardial Failure)) OR (Congestive Heart Failure)) OR ("Heart Failure"[Mesh])) AND ((((Galectin 3) OR (galectin-3)) OR (Gal-3)) OR ("Galectin 3"[Mesh]))**
#6	•••	>	Search: **(((Galectin 3) OR (galectin-3)) OR (Gal-3)) OR ("Galectin 3"[Mesh])**
#5	•••	>	Search: **((Galectin 3) OR (galectin-3)) OR (Gal-3)**
#4	•••	>	Search: **"Galectin 3"[Mesh]** Sort by: **Most Recent**
#3	•••	>	Search: **(((((Heart Failure) OR (Cardiac Failure)) OR (Heart Decompensation)) OR (Myocardial Failure)) OR (Congestive Heart Failure)) OR ("Heart Failure"[Mesh])**
#2	•••	>	Search: **((((Heart Failure) OR (Cardiac Failure)) OR (Heart Decompensation)) OR (Myocardial Failure)) OR (Congestive Heart Failure)**
#1	•••	>	Search: **"Heart Failure"[Mesh]** Sort by: **Most Recent**

The exact search string for Embase

#7. #3 AND #6
#6. #4 OR #5
#5. 'gal-3' OR 'galectin 3'
#4. 'galectin 3'/exp
#3. #1 OR #2
#2. 'heart failure' OR 'cardiac failure' OR 'heart decompensation' OR 'myocardial failure' OR 'congestive heart failure'
#1. 'heart failure'/exp

History Save | Delete | Print view | Export | Email Combine > using (•) And ( ) Or

#7	#3 AND #6
#6	#4 OR #5
#5	**'gal-3'** OR **'galectin 3'**
#4	**'galectin 3'**/exp
#3	#1 OR #2
#2	**'heart failure'** OR **'cardiac failure'** OR **'heart decompensation'** OR **'myocardial failure'** OR **'congestive heart failure'**
#1	**'heart failure'**/exp
